# Radiotherapy-induced cell death activates paracrine HMGB1-TLR2 signaling and accelerates pancreatic carcinoma metastasis

**DOI:** 10.1186/s13046-018-0726-2

**Published:** 2018-04-03

**Authors:** Xuelian Chen, Lirong Zhang, Yujie Jiang, Lian Song, Yanfang Liu, Fang Cheng, Xin Fan, Xiongfeng Cao, Aihua Gong, Dongqing Wang, Haitao Zhu

**Affiliations:** 1 The Affiliated Hospital of Jiangsu University, Zhenjiang, 212001 China; 2 The First People’s Hospital of Zhenjiang, Zhenjiang, 212001 China; 3 Faculty of Science and Engineering, Åbo Akademi University and Turku Centre for Biotechnology, -20520 Turku, FI Finland; 4 School of Medicine, Jiangsu University, Zhenjiang, 212013 China; 5 Department of Radiology, The Affiliated Hospital of Jiangsu University, Jiangsu University, 438 Jiefang Road, Zhenjiang, Jiangsu Province 212013 China

**Keywords:** Pancreatic carcinoma, HMGB1, Metastasis, Radiotherapy

## Abstract

**Background:**

Dying cells after irradiation could promote the repopulation of surviving cancer cells leading to tumor recurrence. We aim to define the role of dying cells in promoting pancreatic cancer cells metastasis following radiotherapy.

**Methods:**

Using the transwell system as the in vitro co-culture model, a small number of untreated pancreatic cancer cells were seeded in the upper chamber, while a larger number of lethally treated pancreatic cancer cells were seeded in the lower chamber. A series of experiments were conducted to investigate the role of dying-cell-derived HMGB1 on the invasion of pancreatic cancer in vitro and cancer metastasis in vivo. We then designed shRNA knockdown and Western blot assays to detect signaling activity.

**Results:**

We found that dying pancreatic cancer cells significantly promote the invasion of pancreatic cancer cells in vitro and cancer metastasis in vivo. HMGB1 gene knockdown attenuated the migration-stimulating effect of irradiated, dying cells on living pancreatic cancer cells. Finally, we showed that dying-cell-derived HMGB1 functions in a paracrine manner to affect cancer-cell migration dependent on acquiring an epithelial-mesenchymal transition (EMT) phenotype and PI3K/pAkt activation. This process is mediated by the receptor for TLR2.

**Conclusion:**

Our study indicates that, during radiotherapy, dying pancreatic cancer cells activate paracrine signaling events that promote the mobility of surviving tumor cells. We suggest a strategy to inhibit HMGB1 for preventing pancreatic carcinoma relapse and metastasis.

**Electronic supplementary material:**

The online version of this article (10.1186/s13046-018-0726-2) contains supplementary material, which is available to authorized users.

## Background

Pancreatic carcinoma (PC) is projected to become the second leading causes of cancer death by the year 2020 [1]. Only 15% of patients presenting with resectable disease have the chance to undergo complete resection, which is the only potentially curative option for PC. The remaining 85% of patients, who have developed locally advanced disease, are treated with either systemic chemotherapy or radiotherapy. Despite advances in radiation and new agents, the five-year survival rate of PC remains no more than 6% [2]. In most pancreatic cancer patients, available therapeutics inhibit the growth of primary tumors but, paradoxically, also promote tumor recurrence and metastasis [3, 4]. Once regional relapse or distant metastatic has occurred, there are no good options to control tumor progression. Thus, it is necessary to investigate the mechanisms underlying this process of radiotherapy accelerating metastasis.

Metastasis cascade consists of a sequence of distinct steps [5]. Initial metastasis requires close collaboration between cancer cells and their environment. Following radiotherapy, the tumor microenvironment is rich in the dying cells and their released soluble factors, including cytokines, growth factors, and adhesion molecules. Recently, several reports have demonstrated that dying-cell-derived soluble factors significantly stimulate the repopulation of resident cancer cells after chemo- or radiotherapy [6–8]. Our earlier research has also confirmed this phenomenon in PC. The repopulation of surviving cancer cells is essential for tumor recurrence and distance metastasis. Huang et al. referred to this as the “Phoenix Rising” [7]. It is clear that radiotherapy could enhance the motility and invasiveness of several cancer cells, including lung, breast and glioma cancer cells [9, 10]. However, the precise mechanisms remain largely unclear. In this study, we plan to explore an initial pro-metastasis factor in the irradiated pancreatic carcinoma microenvironment.

The high-mobility group box 1 (HMGB1) protein, a highly conserved nuclear protein that functions as a chromatin-binding factor, facilitates nucleosome stabilization and regulates gene transcription [11–13]. The functions of HMGB1 in pancreatic cancer are complicated and paradoxical depending on the intracellular or extracellular locations [14]. It acts as an anti-tumor protein when located intracellularly and behaves as a pro-tumor protein when located extracellularly. HMGB1 is actively secreted by inflammatory cells or passively released from dying or stressor cells. HMGB1 is an important component of the tumor microenvironment induced by chemotherapy or radiotherapy. In recent research, extracellular HMGB1, by binding several receptors, including the receptor for advanced glycation end products (RAGE) and Toll-like receptors TLR-2, TLR-4, and TLR-9, has been shown to trigger the key signaling pathways involved in the regulation of pancreatic carcinoma growth, autophagy, immunogenic cell death, and resistance to chemotherapy [15, 16]. Several reports have also revealed that HMGB1 is involved in the metastatic phenotype of pancreatic cancer [17, 18].

However, the precise mechanisms for HMGB1 regulating pancreatic-cancer metastasis are still to be elucidated. EMT is closely linked to the induction of metastasis [19, 20]. Cancer cells undergoing EMT acquire invasive and metastatic properties. In response to the stresses, including hypoxia, chemo- or radiotherapy, and inflammation, the EMT program can be triggered by multi-regulatory networks that involve soluble factors (transforming growth factor-β [TGF-β] and epidermal growth factor [EGF]) and their associated signaling proteins (Wnt, Notch, PI3K/Akt). HMGB1 has been implicated in EMT in several tumor models [21, 22]. However, the association of dying-cell derived HMGB1 and EMT in pancreatic-cancer metastasis has rarely been investigated, and the underlying molecular mechanism remains unclear. Therefore, we are interested if dying pancreatic-cancer cells undergoing apoptosis during radiotherapy activate HMGB1-mediated paracrine signaling events that promote the migration of surviving tumor cells.

To test this hypothesis, we used the transwell co-culture model and the results suggest that dying pancreatic cancer cells significantly promote pancreatic cancer cell invasion in vitro and metastasis in vivo. We found that inhibiting HMGB1 by down-regulating HMGB1 in irradiated cancer cells or by adding an HMGB1 inhibitor in the supernatant attenuated the pancreatic cancer cell migration. We found that HMGB1 functions in a paracrine manner to affect cell migration and a PI3K/pAkt-EMT signaling axis is involved in the process.

## Methods

### Cell culture

Human pancreatic-cancer cell lines were obtained from the Cell Bank of the China Academy of Sciences (Shanghai, China). Panc-1 cells, a human pancreatic carcinoma of ductal cell origin, were maintained in Dulbecco’s modified Eagle’s medium (DMEM) supplemented with 10% fetal bovine serum (FBS, Gibco, NY, USA), 100 U/mL penicillin and 100 U/mL streptomycin. SW1990 cells, a human pancreatic metastatic-site-derived adenocarcinoma, were maintained in L-15 medium supplemented with 10% FBS, 100 U/mL penicillin, and 100 U/mL streptomycin. The cells were maintained in a humidified atmosphere with 5% CO_2_ at 37 °C and passaged with 0.25% trypsin/EDTA every 3 days.

### Irradiation and in vitro co-culture system of cancer cells

2 × 10^6^ cells (pancreatic cancer cells and human fibroblast cells) cultured in 6 cm dishes were irradiated with various doses of irradiation (0, 4, 8, and 12 Gy) using a linear accelerator (Turebeam-STX, Varian, USA). The dose rate of the machine is about 4 Gy/min. Irradiated cells were immediately trypsinized and seeded into the lower chamber of the transwell system (the transwell filter inserted using a 6.5 mm diameter with a pore size of 5 μm; Corning, Inc.) as feeders at a density of 1 × 10^5^ cells per well in DMEM containing 10% FBS. 5 × 10^4^ pancreatic cancer cells were seeded into the upper chamber of the transwell system, which was pre-coated with 60 μl Matrigel (BD Bioscience, NY, USA) at indicated time points after seeding the feeders.

### Conditioned medium preparation

Equal numbers of tumor cells were seeded into cell-culture dishes overnight. The culture medium was replaced by a low-serum medium (2% FBS) before irradiation. A total of 12 h after irradiation, the culture medium was collected, centrifuged at 3000 rpm for 10 min, filtered with a 0.22 mm filter unit to remove cellular debris, and stored at − 80 °C until use.

### ELISA assay

To measure HMGB1 release, pancreatic cancer cells were grown in 6 cm dishes to 80% confluence and then irradiated with various doses of irradiation (0, 4, 8, and 12 Gy). Supernatants were collected at the indicated times (0, 6, 12, 24, and 48 h) after initiation. HMGB1 levels in the supernatants were measured using the ELISA kit (Chondrex, USA).

### Annexin V/PI apoptosis assay

The apoptotic ratios of cancer cells were determined using Annexin V/ propidium iodide (PI) apoptosis detection kits (Invitrogen, Shanghai, China). Apoptotic cells were analyzed based on PI and Annexin V staining as described previously [23]. Briefly, after treatment with various doses of irradiation (0, 4, 8 and 12 Gy), the cells were collected and washed twice with ice-cold PBS buffer, re-suspended in 100 μL of 1 × Annexin binding buffer, incubated with 5 μL of Annexin V conjugated to FITC and 1 μL PI (100 mg/mL) for 15 min at room temperature, then resuspended in 400 μL of 1 × Annexin binding buffer and analyzed by flow cytometry. The experiments were repeated three times.

### Flow cytometry analysis

Cluster of differentiation 133(CD133) staining was carried out as described previously [24]. In brief, 5 × 10^6^ pancreatic cancer cells were harvested, disaggregated into a single-cell suspension, and incubated with 2 mg/ml mouse anti-human CD133/PE antibody for 30 min at 4 °C in the dark. After incubation, the samples were washed with PBS and analyzed using FACS AriaII (Becton Dickinson, USA).

### Drug treatment

rhHMGB1 (HMGBiotech, Germany) was dissolved in distilled water to make a 1000 ng/ml stock solution. When the cells grown to 80% confluence, various concentrations of rhHMGB1 (50 ng/mL, 100 ng/mL, 150 ng/mL, and 200 ng/mL) were added directly to the media for the indicated time. The treated cells were subjected to the following experiments.

MK2206, which inhibits phosphorylation of AKT, was purchased from MCE (USA) and dissolved in dimethylsulfoxide (DMSO). Cells were grown to 80% confluence, treated with 2 μM MK2206 for the indicated time and subjected to the following experiments.

### shRNA knockdown

Pancreatic cancer cells were seeded in six-well plates at a density of 1 × 10^5^ cells/well to achieve a confluence of 70–80% overnight. Next, RAGE-shRNA, TLR-2-shRNA, TLR-4-shRNA, CD24-shRNA, Zeb1-shRNA, and negative control shRNA (Suzhou Ribo Life Science CO., Ltd, Suzhou, China) were transfected into cells, respectively, using a transfection reagent (lipofectamine 2000, Invitrogen), following the manufacturer’s instructions.

To establish the stable sh-HMGB1 cancer cells, a lentiviral packaging kit was purchased from Open GeneCopoeia. Lentivirus carrying HMGB1-shRNA was packaged in 293 T cells and concentrated from the supernatant, following the manufacturer’s instructions. Stable cell lines were established by infecting lentivirus into pancreatic cancer cells followed by puromycin (1 μg/ml) selection for 10–14 days. These established stable cell lines were maintained in DMEM containing 10% FBS and puromycin (0.75 μg/ml) for further experiments.

The following specific shRNA sequences were used:

- The human HMGB1-shRNA1:
- 5’-CCGGCCCAGATGCTTCAGTCAACTTCTCGAGAAGTTGACTGAAGCATCTGGGTTTTT-3’
- The human HMGB1-shRNA2:
- 5’-CCGGCCGTTATGAAAGAGAAATGAACTCGAGTTCATTTCTCTTTCATAACGGTTTTT-3’
- The human RAGE-shRNA1:
- 5’-CCGGCGAGTCCGTGTCTACCAGATTCTCGAGAATCTGGTAGACACGGACTCGTTTTTG-3’
- The human RAGE-shRNA2:
- 5’-CCGGGAAGGGAGTATCTGTGAAGGACTCGAGTCCTTCACAGATACTCCCTTCTTTTTG-3’
- The human TLR2-shRNA1:
- 5’-CCGGGCATCTGATAATGACAGAGTTCTCGAGAACTCTGTCATTATCAGATGCTTTTTG-3’
- The human TLR2-shRNA2:
- 5’-CCGGGCACACGAATACACAGTGTAACTCGAGTTACACTGTGTATTCGTGTGCTTTTTG-3’
- The human TLR4-shRNA1:
- 5’-CCGGCCAAGTAGTCTAGCTTTCTTACTCGAGTAAGAAAGCTAGACTACTTGGTTTTTG-3’
- The human TLR4-shRNA2:
- 5’-CCGGCCCTGCTGGATGGTAAATCATCTCGAGATGATTTACCATCCAGCAGGGTTTTTG-3’
- The human CD24-shRNA1:
- 5’-CCGGCTTCTGCATCTCTACTCTTAACTCGAGTTAAGAGTAGAGATGCAGAAGTTTTTG-3’
- The human CD24-shRNA2:
- 5’-CCGGTGCTCCTACCCACGCAGATTTCTCGAGAAATCTGCGTGGGTAGGAGCATTTTTG-3’
- The human Zeb1-shRNA1:
- 5’-CCGGCCTCTCTGAAAGAACACATTACTCGAGTAATGTGTTCTTTCAGAGAGGTTTTT-3’
- The human Zeb1-shRNA2:
- 5’-CCGGCCTACCACTGGATGTAGTAAACTCGAGTTTACTACATCCAGTGGTAGGTTTTTG-3’

### Western blot analysis

Protein concentrations were determined using the bicinchoninic acid assay (BCA) method. Western blot assay was carried out as described previously [25]. Antibodies against HMGB1 were purchased from Abcam Company. Antibodies against pAkt, vimentin, were purchased from Santa Cruz Biotechnology. Antibodies against Caspase-3, PI3k, Akt, E-cadherin, and N-cadherin, were obtained from Cell Signaling Technology, Inc. (Boston, USA). Anti-β-Tubulin was obtained from Abcam Company (Cambridge, USA). The secondary antibody preparations, either anti-rabbit or anti-mouse, were purchased from Boster biotechnology company (Wuhan, China).

### Real-time polymerase chain reaction (PCR)

Real-time quantitative PCR was carried out with SYBR Green qPCR SuperMix (Bio-Rad) using the CFX-96 system (Bio-Rad). Total cellular RNA was isolated using TRIzol reagent, and cDNA was synthesized from 1 μg of the total RNA using oligo(dT) and murine Moloney leukemia virus reverse transcriptase (Toyobo, Japan). Relative expression levels of the genes were calculated using the 2 − ΔΔCT method. The obtained results were normalized to GAPDH levels. The following gene-specific primers were used:

- Snail (5’ACC CCA CAT CCT TCT CAC TG-3′, 5’-TAC AAA AAC CCA CGC AGA CA-3′).
- Slug (5’ACA CAC ACA CAC CCA CAG AG-3′, 5′-AAA TGA TTT GGC AGC AAT GT-3′).
- Zeb1 (5’-GCA CAA CCA AGT GCA GAA GA-3′, 5’-CAT TTG CAG ATT GAG GCT GA-3′).

### Xenograft tumor models

Animal studies were approved by the Committee on the Use of Live Animals for Teaching and Research of the Jiangsu University. Female BALB/c nude mice (purchased from The Compare Medicine Center, Yangzhou University, China), aged 6 weeks, were maintained under standard conditions according to institutional guidelines.

Panc-1 cells were continuously cultured with the control supernatant, N-S, N-S + EP, HMGB1^−/−^ N-S, and rhHMGB1 (150 ng/ml), for 2 weeks. Half of the culture medium was replaced by a fresh medium containing the above-mentioned materials every day for 2 weeks. Next, 2 × 10^6^ cancer cells were injected into the tail vein of each mouse. The metastatic lung foci and lymph node were accessed by computed tomography (CT) scanning and histological haematoxylin and eosin (HE) staining. The metastatic area was quantified for each mouse, with each group containing a minimum of three mice.

### In vivo CT scanning

CT scanning was performed by 256 slices CT (Revolution CT, GE Company, America). For CT scans, animals were anesthetized by inhalation of a mixture of oxygen and 4% isoflurane. The mice were positioned in the scanner bed in the prone position with the imaging field of view (FOV) centered at the abdomen. CT scanning parameters were set as follows: pitch = 0.984:1; speed = 39.37 mm/rot; detector coverage = 40 mm; rotation time = 0.7 s; helical thickness = 0.625 mm; interval = 0.625; FOV = 96 mm × 96 mm; and matrix = 512 × 512.

### HE staining and immunohistochemistry

Paraffin-embedded tissues were sectioned for HE staining and immunohistochemical (IHC) analysis. For HE staining, slides were deparaffinized, hydrated, and stained with hematoxylin for 1 min. After rinsing, the slides were stained with eosin for 1 min, rinsed, and sealed with cover slips using Permount™ Mounting Medium. For IHC, samples were fixed in 10% formalin and embedded in paraffin wax. Next, 3 mm sections were cut from the paraffin blocks for IHC analysis. The sections were stained with mouse anti-cleaved caspase-3 (1:500), mouse anti-MMP-9 (1:500), and anti-HMGB1 (1:500) at 4 °C overnight. All the sections were cover slipped with neutral balsam and viewed under an Olympus microscope and analyzed using Image J software. The final result for each case was the average score of all visual fields.

### Immunofluorescence analyses

Tumor sections were analyzed following standard haematoxylin and eosin procedures or immunostaining protocols as previously reported [25]. Nikon microscopy and NIS element software were used for imaging and quantification. Primary antibodies used are as follows: anti-cleaved caspase-3 (1:500); and anti-HMGB1 (1:1000). Quantification of images was performed by assessing 20× high-power fields per slide in a blinded manner.

### Patient selection

A total of 40 patients with histologically or medicine-imaging confirmed pancreatic carcinoma that underwent radiotherapy between 1 January 1 2013 and 30 December 30 2016 at the Affiliated Hospital of Jiangsu University were enrolled in this study. Information about lymph node, distant metastasis, and disease-related death were obtained from medical records or telephone interviews. Radiological staging was used to access the response to therapy according to standard response-evaluation criteria in solid tumors. The clinic pathological characteristic of the 40 patients is summarized in Table 1. Written informed consent was obtained from each patient. The study protocol was approved by the Ethics Committee of the Affiliated Hospital of Jiangsu University.

**Table 1:** Summary of clinical characteristics in 40 patients with pancreatic cancer

	Number	Percentage	HMGB1^high^ [*n* (%)]
Patients	40		32 (80)
Gender			
Male	28	70	23 (57.5)
Female	12	30	9 (22.5)
Age			
Median	56		
Range	34–75		
≤ 50 y	16	40	11 (27.5)
>50 y	24	60	21 (52.5)
TNM stage			
Stage I	3	7.5	1 (2.5)
Stage II	14	35	7 (17.5)
Stage III	17	42.5	16 (40)
Stage IV	6	15	6 (15)
Lymph nodes			
Positive	9	22.5	8 (20)
Negative	31	77.5	6 (15)
Distance metastasis			
Positive	6	15	6 (15)
Negative	34	85	8 (22.5)
Response to therapy			
Partial response + stable disease	6	15	2 (5)
Progressive disease	29	72.5	25 (62.5)
Not assessed	5	12.5	

Blood samples were collected prospectively before the start of radiotherapy and then weekly during therapy until the first radiologic staging after two months. They were centrifuged for 15 min at 3000 g within 2 h of collection. The resulting sera were aliquoted into microtubes and either immediately frozen at − 80 °C or previously stabilized with 10 mM EDTA (pH 8) for HMGB1 measurement.

The Cancer Genome Atlas (TCGA) database (https://xenabrowser.net/datapages/?cohort=TCGA%20Pancreatic%20Cancer%20(PAAD); TCGA BRCA exp. HiSeqV2PANCAN-2014-05-02), including 168 pancreatic carcinoma patient specimens, was utilized to further analyze the relationship between HMGB1, Caspase-3, and EMT-related proteins. The association of HMGB1 expression level with overall survival, metastasis-free survival, and recurrence was also analyzed. High and low groups were defined as above and below the mean, respectively.

### Statistical analysis

All data are presented as the mean ± SEM (standard error of the mean). Linear regression and F-tests were used to determine the significance of the TCGA data. Kaplan–Meier analysis was used to estimate overall survival rate of the enrolled patients. The significances of differences between groups were analyzed using Student’s t-tests or one-way ANOVA. Values of *p* < 0.05 were considered significant. All the experiments were repeated at least three times.

## Results

### X-ray irradiation of human pancreatic cancer cells promote tumor cell invasion in vitro

**Fig. 1:**
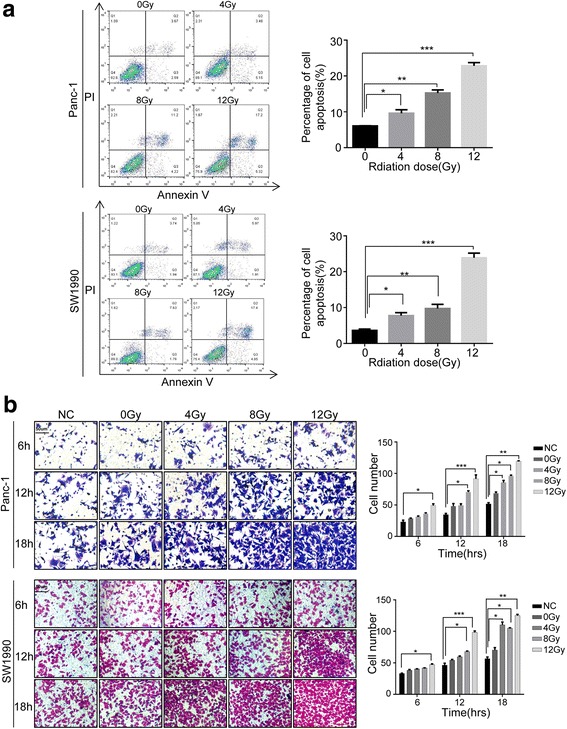
Irradiation-induced cell death promotes cancer-cell metastasis in vitro. **a** Annexin V /PI for the apoptosis cancer cell percentage in Panc-1 and SW1990 cells treated with various doses X-ray (0, 4, 8, and 12 Gy). The apoptosis cancer cell increased in a dose-dependent manner. **b** Irradiated Panc-1 and SW1990 cells stimulated untreated cancer cells’ metastasis in a dose- and time-dependent manner. Varied-dose X-ray-treated (0, 4, 8, and 12 Gy) Panc-1 and SW1990 cells seeded in the lower chamber and untreated Panc-1 and SW1990 cells in the upper chamber co-cultured in the transwell system for the indicated time (6, 12, and 18 h). Imagings were taken by electron microscope. Magnification, × 20. Experiments were repeated three times and the data were expressed as mean ± SEM. *p < 0.05, **p < 0.01, ***p < 0.001

First, to achieve significant cell death by x-ray irradiation in our in vitro model, we optimized the irradiation doses on Panc-1 and SW1990 cells by examining cell apoptosis after irradiation via FACS analysis. According to previous research and our pilot experiment, we chose 12 Gy as the maximum irradiation dose that can mimic the in vivo maximum radiation dose. The results showed a significant increase of apoptotic cell numbers after irradiation in a dose-dependent manner, with more apoptotic cells in the 12 Gy group (Panc-1 cells, 22.83 ± 0.74%; SW1990, 23.96 ± 0.83%) than in the 8 Gy group (Panc-1 cells, 15.25 ± 0.69%; SW1990, 10.06 ± 0.17%) (Fig. 1a). Based on these findings, we used 12 Gy to induce apoptosis in Panc-1 and SW1990 cells in the following experiments.

Next, we examined the invasion-promoting effect of irradiated pancreatic cancer cells. 1 × 10^5^/well Panc-1 and SW1990 cells were seeded into the lower chamber of the transwell system and treated with gradient doses of irradiation (4 Gy, 8 Gy, and 12 Gy). 12 h post irradiation, 5 × 10^4^/well Panc-1 and SW1990 cells were seeded onto the upper chamber of the transwell invasion system, and co-incubated in DMEM containing 10% FBS for the indicated times (6 h, 12 h, and 18 h). The number of cancer cells passed through the membrane was measured and compared with cancer cells cultured alone (NC) or co-cultured with untreated cancer cells (0 Gy). As shown in Fig. 1b, cancer cells in the upper chamber passed through the membrane in a dose- and time-dependent manner, with the peak at 12 Gy for 18 h. Also, a greater number of cancer cells in the radiated feeder-cells groups (12 Gy irradiation cells) migrated through the chamber membranes compared to their corresponding control groups for 18 h (Panc-1 cancer cells), about 2 times higher than cultured alone cells and 1.5 times higher than co-cultured with untreated cancer cells; SW1990 cancer cells, about 2.5 times higher than cultured alone cells and 2 times higher than co-cultured with untreated cancer cells). As radiotherapy can induce the death of both cancer cells and surrounding stromal cells in solid tumors, we next wanted to examine whether dying fibroblast cells could promote cancer cells’ transwell migration. As expected, lethally irradiated human fibroblast cells stimulated cancer cell invasion significantly in vitro (Additional file 1: Figure S1).

### HMGB1 is released from the dying cells after irradiation in vitro and in vivo

**Fig. 2:**
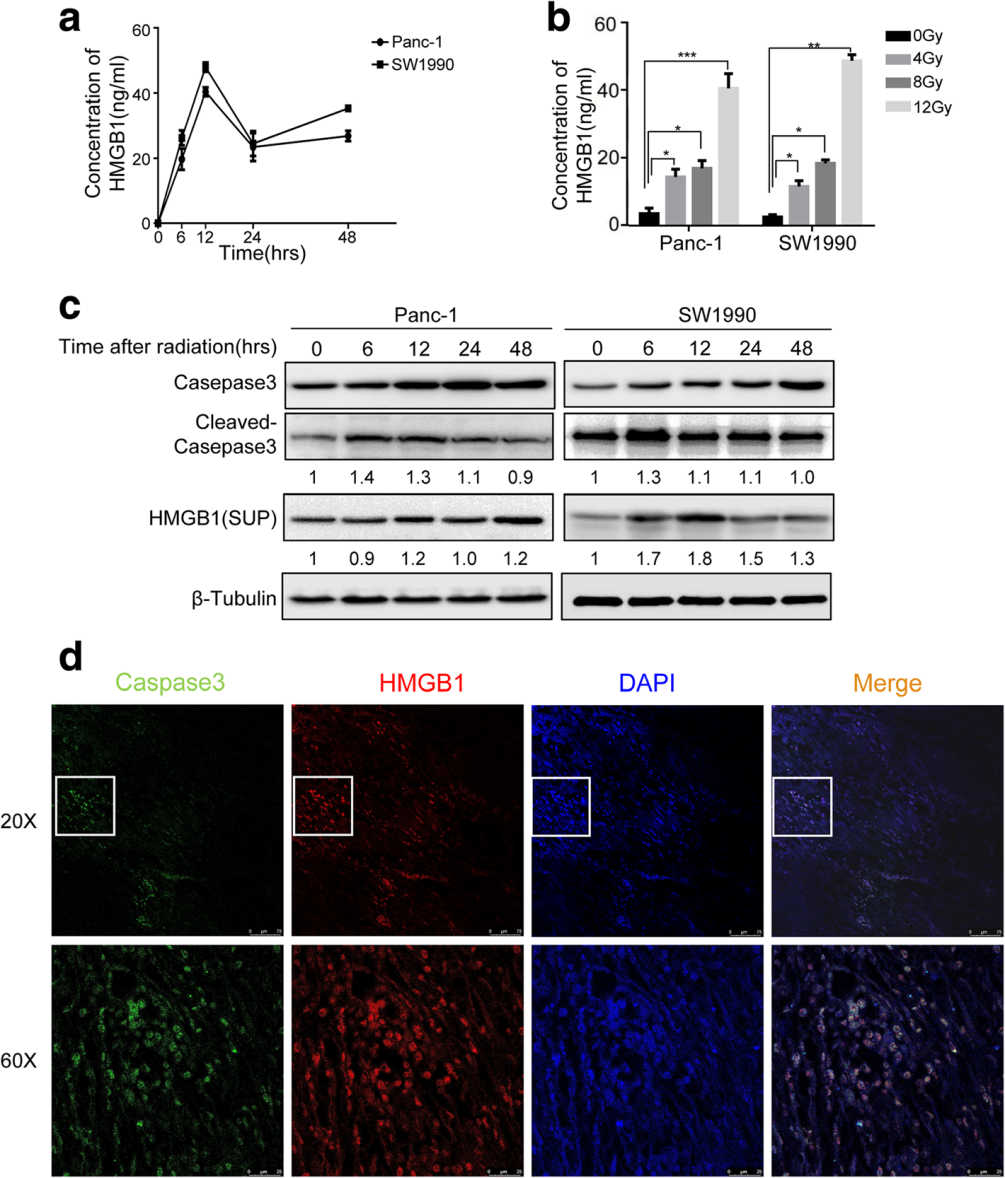
HMGB1 released from the dying cells in vitro and in vivo. **a** Release of HMGB1 in the supernatants by 12 Gy-treated Panc-1 and SW1990 was measured by ELISA at the indicated time points (0, 6, 12, 24, and 48 h). **b** Release of HMGB1 in the supernatants by various radiation doses (0, 4, 8, and 12Gy) treated Panc-1 and SW1990 cells were measured by ELISA. **c** Western blot analyzing the correlative protein level of Cleaved Caspase-3 and HMGB1 in the supernatants at various time following 12 Gy-treated Panc-1 and SW1990. β-Tubulin was a loading control. **d** Confocal immunofluorescence analysis demonstrating co-localization of Caspase3 positive cells (green) and HMGB1-positive cells (red) in the xenograft tumors following radiotherapy. Magnification: × 20 and × 60. Experiments were repeated three times and the data were expressed as mean ± SEM. *p < 0.05, **p < 0.01, ***p < 0.001

Next, we tested whether HMGB1 is released from apoptotic cells after radiotherapy. We first examined the release of HMGB1 from the pancreatic cancer cells into the medium after irradiation. ELISA results showed that the level of HMGB1 released into the cell supernatants depends on irradiation dose, with the highest concentration observed at 12 Gy for 12 h (Panc-1, 40.51 ± 3.57 ng/ml and SW1990, 48.67 ± 1.48 ng/ml; Fig. 2a and b). We next investigated the correlation of irradiation-induced apoptosis and the release of the HMGB1. The results demonstrated that the expression of cleaved caspase-3, a key apoptotic maker, peaked at 6 h and gradually reduced thereafter, which was closely followed by HMGB1 release from 12 to 48 h after 12 Gy X-ray irradiation (Fig. 2c). To further confirm the link between HMGB1 and apoptosis, we established a xenograft tumor model by injecting 1 × 10^5^ Panc-1 cancer cells subcutaneously into the right flank of the nude mouse. When the tumor reached the 1mm^3^, the mouse received 4 Gy radiation. 1 week after treatment, the tumor tissue was removed to analyze the co-localization of HMGB1 and cleaved Caspase-3 by immunofluorescence. The results showed that HMGB1 is localized adjacent to cleaved Caspase-3 positive cancer cells (Fig. 2d). Altogether, these findings suggested that HMGB1 is released from apoptotic cells after irradiation in vitro and in vivo.

### Dying cell-derived HMGB1 regulates tumor cell migration through TLR2 in vitro

**Fig. 3:**
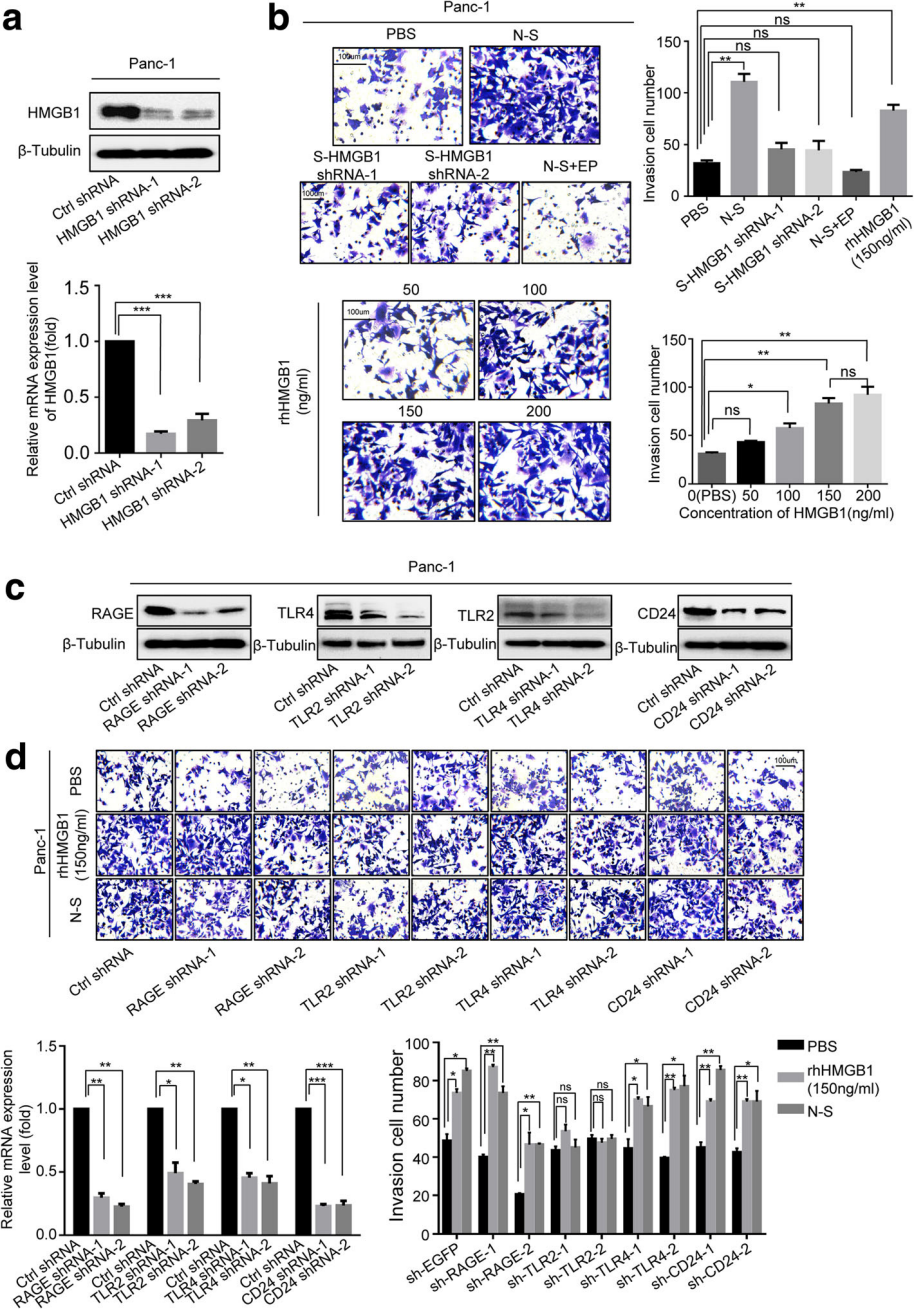
Dying-cell-derived HMGB1 regulates tumor-cell metastasis through TLR2 in vitro. **a** Western blot showing shRNA-knockdown efficiency HMGB1 in Panc-1 cancer cells. β-Tubulin was a loading control. **b** Panc-1 cells’ migration ability following being treated with N-S (supernatants from the parental cancer cells following 12 Gy radiation), HMGB1^−/−^-S (supernatants from the HMGBl knockdown cancer cells following 12 Gy radiation), N-S + EP (HMGB1 inhibitor), and different concentrations of rhHMGB1 (50, 100, 150, and 200 ng/mL) for 12 h was analyzed by transwell invasion assays. Migratory cells were counted in at least three to four randomly-selected microscopic fields and the results are expressed as the mean ± SEM of migratory cells per microscopic field. Magnification: × 20. **c** Western blot showing shRNA-knockdown efficiency of RAGE, TLR2, TLR4, and CD24 in Panc-1 cells, respectively. β-Tubulin was a loading control. **d** The invasion ability of Panc-1 cells following knockdown: the receptor, treated with PBS, N-S, rhHMGB1 (150 ng/mL), was accessed by transwell assay. TLR2, rather than other receptors, mediated the HMGB1-induced Panc-1 cells’ metastasis. Magnification: × 20. Experiments were repeated three times and the data were expressed as mean ± SEM. *p < 0.05, **p < 0.01, ***p < 0.001

To further confirm HMGB1 release from the dying cancer cells (required to promote cancer cell invasion), we performed HMGB1 stable knockdown in Panc-1 feeder cells (HMGB1^-/-^Panc-1) using shRNA technology and confirmed the silencing efficiency by Western blot (Fig. 3a). The control Panc-1 cells and HMGB1^-/-  ^Panc-1 were irradiated with 12 Gy X-ray and the supernatant (the control Panc-1 cell-derived supernatant was named N-S and HMGB1^-/-^Panc-1 cell-derived supernatant was named HMGB1^−/−^-S) was collected for further experimental use.

To test the functional role for HMGB1 in promoting pancreatic-cancer-cell invasion, we treated Panc-1 cells with PBS (control), N-S, HMGB1^−/−^-S, N-S + EP (Ethyl pyruvate, HMGB1 inhibitor, 1 mg/ml), and different concentrations of recombinant human HMGB1 (rhHMGB1, 50 ng/mL, 100 ng/mL, 150 ng/mL, and 200 ng/mL) for 12 h, respectively. As shown in Fig. 3b, the number of living cancer cells that passed through the membrane under N-S- and rhHMGB1-treated conditions was much higher than the number of the cells for the control group. Meanwhile, 100 ng/mL-concentration rhHMGB1 significantly promoted cell invasion. As there was no significant difference between the 150 ng/mL- and 200 ng/mL-treated group, the peak migration was induced by 150 ng/mL concentration. Compared to the N-S group, the number of cancer cells that passed through the membrane under HMGB1^−/−^-S- and N-S + EP-treated groups was much lower. Moreover, we noticed that the number of migrating cancer cells in the N-S + EP-treated group was much less than in the HMGB1^−/−^-S-treated group, indicating EP as an additional mechanism in inhibiting cell invasion. These results supported the hypothesis that dying cell-derived HMGB1 promotes the invasion potential of living cancer cells.

HMGB1 has been shown to promote cancer cells’ proliferation. To avoid the effect of dying cell-derived HMGB1 on proliferation, we treated Panc-1 cells with PBS (control), N-S, HMGB1^−/−^-S, N-S + EP, and rhHMGB1 (150 ng/mL) for different time. CCK8 assay demonstrated that there was no significant difference in cell proliferation in each group for the indicated time (Additional file 2: Figure S2A). Western blot also confirmed that the expression level of Ki67 was not significantly different between each group (Additional file 2: Figure S2B). These results indicate that HMGB1 promoted the metastasis and invasion of pancreatic cancer cells without the involvement of proliferation in the indicated time.

To determine which HMGB1 receptor(s) is involved in mediating cell invasion, we silenced four well-recognized HMGB1 receptors (RAGE, TLR2, TLR4, and CD24) individually by transiently transfecting specific shRNA oligos in Panc-1 cancer cells for 48 h (Fig. 3c). The cells were subsequently treated with PBS (control), N-S, or 150 ng/mL rhHMGB1 for 12 h. We found that silencing of TLR2, but not RAGE, TLR4, or CD24, inhibited the migratory ability of the living cancer cells (Fig. 3d), indicating that HMGB1 regulates tumor cell invasion through TLR2 in vitro.

### PI3K/Akt pathway and EMT program are involved in HMGB1-mediated cell invasion

**Fig. 4:**
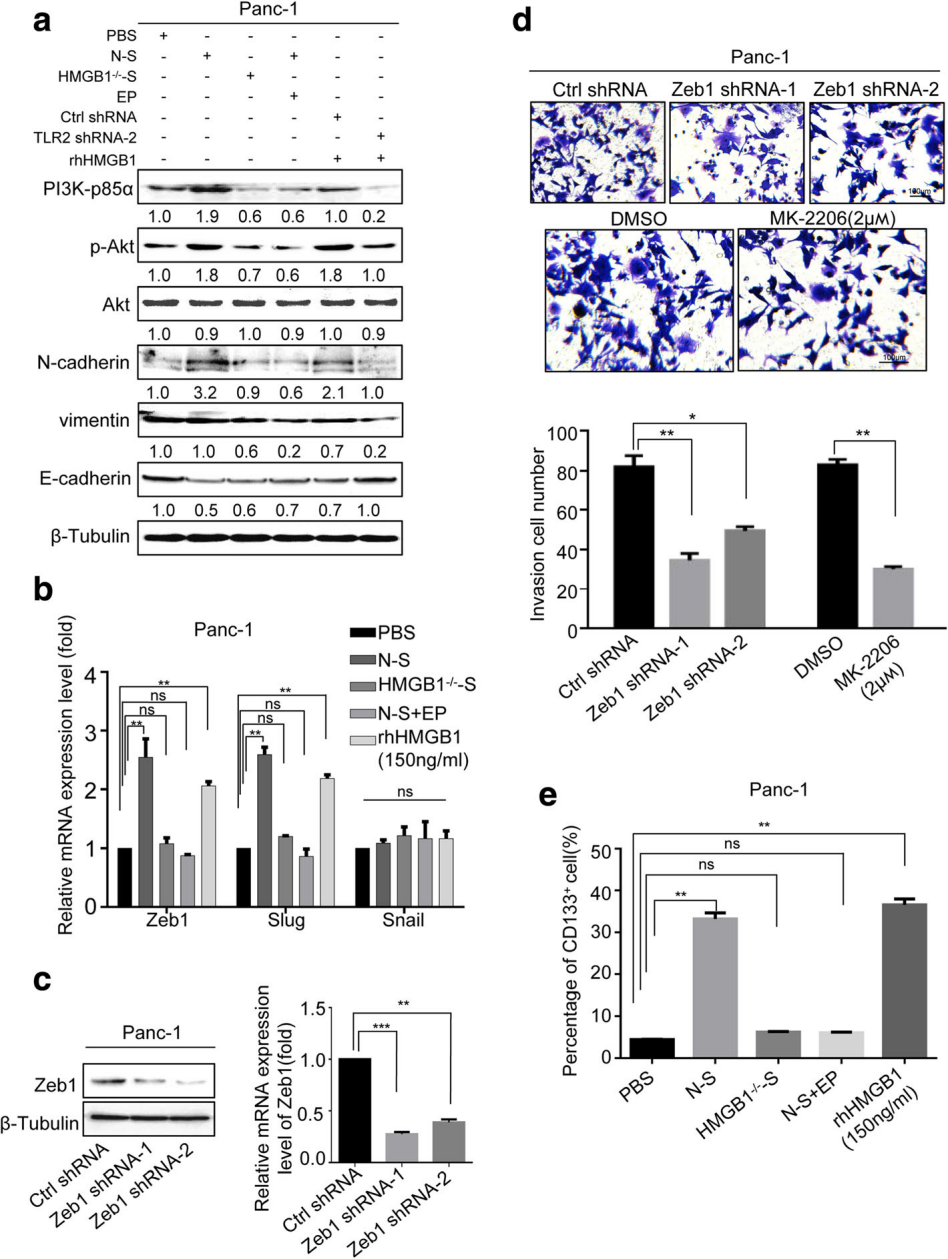
PI3K/Akt pathway and EMT program was responsible for the dying cell derived HMGB1 mediating metastasis. **a** Western blot analyzing the expression of PI3K-p85α, p-Akt, N-cadherin, vimentin, and E-cadherin in Panc-1 cells treated with PBS, N-S, HMGB1^−/−^-S, N-S + EP, and rhHMGB1 (150 ng/mL). β-Tubulin was a loading control. **b** RT-PCR analyzing the expression of EMT-related transcriptional factors Zeb1, Slug, and Snail in Panc-1 cells treated with PBS, N-S, HMGB1^−/−^-S, N-S + EP, and rhHMGB1 (150 ng/mL). GAPDH was a loading control. **c** Western blot showing the shRNA-knockdown efficiency of Zeb1. β-Tubulin was a loading control. **d** The invasion ability of Panc-1 cells following Zeb1 knockdown or treated with DMSO: MK2206 (AKT phosphorylation inhibitor) in the presence of rhHMGB1 (150 ng/mL) was detected by transwell assay. Magnification: × 20. Experiments were repeated three times and the data were expressed as mean ± SEM. *p < 0.05, **p < 0.01, ***p < 0.001. **e** Flow cytometry analyzing the percentage of CD133 cancer cells in Panc-1 cells treated with PBS, N-S, HMGB1^−/−^-S, N-S+EP, and rhHMGB1 (150ng/mL)

As HMGB1 has been reported to induce PI3K/AKT pathway and subsequent EMT program, we hypothesize that HMGB1/TLR2 signaling may activate downstream PI3K/Akt/EMT signaling to accelerate cell migration. To test this hypothesis, we treated Panc-1 cells with PBS (control), N-S, HMGB1^−/−^-S, N-S + EP, or 150 ng/mL rhHMGB1 for 12 h, respectively. Western blot (Fig. 4a) showed that N-S and rhHMGB1 significantly increased the expression PI3K-p85α (a subunit of PI3K), phosphorylated Akt (Ser473), mesenchymal markers N-cadherin and vimentin, and reduced the expression of the epithelial marker E-cadherin. Compared to control samples, HMGB1^−/−^-S and N-S + EP samples induced significantly lower levels of PI3K/Akt/EMT signaling (Fig. 4a), suggesting that HMGB1 released from dying cells triggers PI3K/Akt/EMT signaling.

To assess the transcription factors involved in this EMT signaling, we sequentially analyzed gene expression of EMT-related transcriptional factors (Zeb1, Slug, and Snail) by RT-PCR analysis. The data showed N-S and rhHMGB1 treatment dramatically induced Zeb1 and Slug expression but did not enhance Snail gene expression in a significant level (Fig. 4b). HMGB1 inhibition in the feeder supernatant significantly decreased the up-regulation effect of Zeb1 and Slug on cancer cells (Fig. 4b).

We next further determined whether Akt and Zeb1 are involved in the stimulatory effect of cancer-cell invasion. MK-2206 was used to treat Panc-1 cancer cells to inhibit the phosphorylation of Akt. We also performed Zeb1 stable knockdown in Panc-1 cells using shRNA technology and confirmed the silencing efficiency by Western blot (Fig. 4c). Of note, either knockdown Zeb1 or inhibit the phosphorylation of Akt activity, HMGB1 induced the migratory capacity of cancer cells was abrogated (Fig. 4d).

We also found that N-S and rhHMGB1 treatment induced cancer cells to express cancer stem cell marker CD133 (Fig. 4e), which can be inhibited by either silencing the HMGB1 gene in the secreting cells (HMGB1^−/−^-S group) or adding HMGB1 inhibitor in the supernatant (N-S + EP group).

### Dying-cell derived HMGB1 regulate tumor-cell metastasis in vivo

**Fig. 5:**
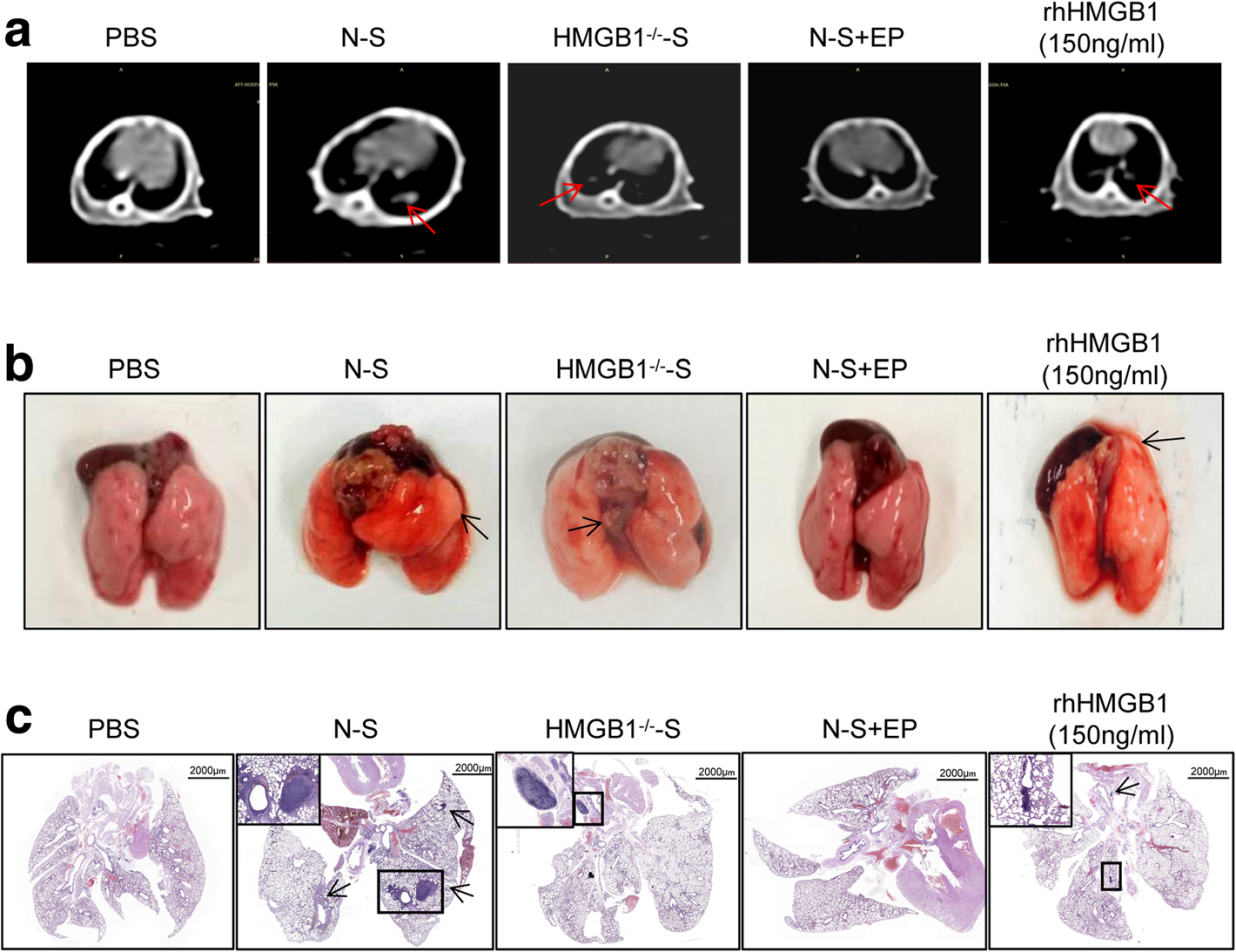
Dying cell derived HMGB1 regulate tumor cell metastasis in vivo. **a** Selected images from the CT dataset of differently treated Panc-1 tumor model groups. Soft-tissue density nodules are clearly visible (arrowheads) in axial plane scanning. **b** Representative lung and mediastinal gross specimen from nude mice are shown. **c** HE staining was used to verify the lung and lymph node metastases foci. Magnification: × 4 and × 20

We next tested whether the in vitro observation of dying cell-released HMGB1 promoting cancer cell invasion could be replicated in vivo. We therefore examined the metastasis of pancreatic cancer cells in nude mice by injecting 1 × 10^6^ Panc-1 cells cultured with PBS, N-S, HMGB1^−/−^-S, N-S + EP, or rhHMGB1 (150 ng/ml) into the mouse tail vein. To measure the metastatic lung foci and lymph node formation, we used computed tomography (CT) to detect metastases 2 weeks after the injection of the Panc-1 cells. In the N-S, HMGB1^−/−^-S, and rhHMGB1 groups, animals exhibited soft-tissue density lesions in the lung acquisition by axial scanning. In the remaining control and N-S + EP groups, mice did not present with lung metastases by CT (Fig. 5a). Following the CT scanning, mice were sacrificed and lungs were harvested for histological analysis. From the HE staining (Fig. 5b), we found that N-S, HMGB1^−/−^-S, N-S + EP, and rhHMGB1 groups formed metastatic lung foci. However, cancer cells exposed to N-S and rhHMGB1 showed significantly more and larger metastatic foci than HMGB1^-/-^-S and N-S + EP groups (Fig. 5b and c and Table 2). Taken together, these results indicate that dying-cell-derived HMGB1 may promote pancreatic cancer metastasis.

**Table 2:** Numbers of animals with lymph-gland metastases and lungs nodes in the different treatment groups

Group	PBS	N-S	sh-HMGB1-S	N-S + EP	rhHMGB1 (150 ng/ml)
Numbers of animals	4	4	4	4	4
Lung nodules	0/4 (0%)	3/4 (75%)	1/4 (25%)	1/4 (25%)	3/4 (75%)
Lymph nodes	1/4 (25%)	2/4 (75%)	0/4 (0%)	0/4 (0%)	1/4 (25%)

### The clinical relevance of radiotherapy-induced HMGB1-TLR2 signaling in pancreatic-cancer metastasis

**Fig. 6:**
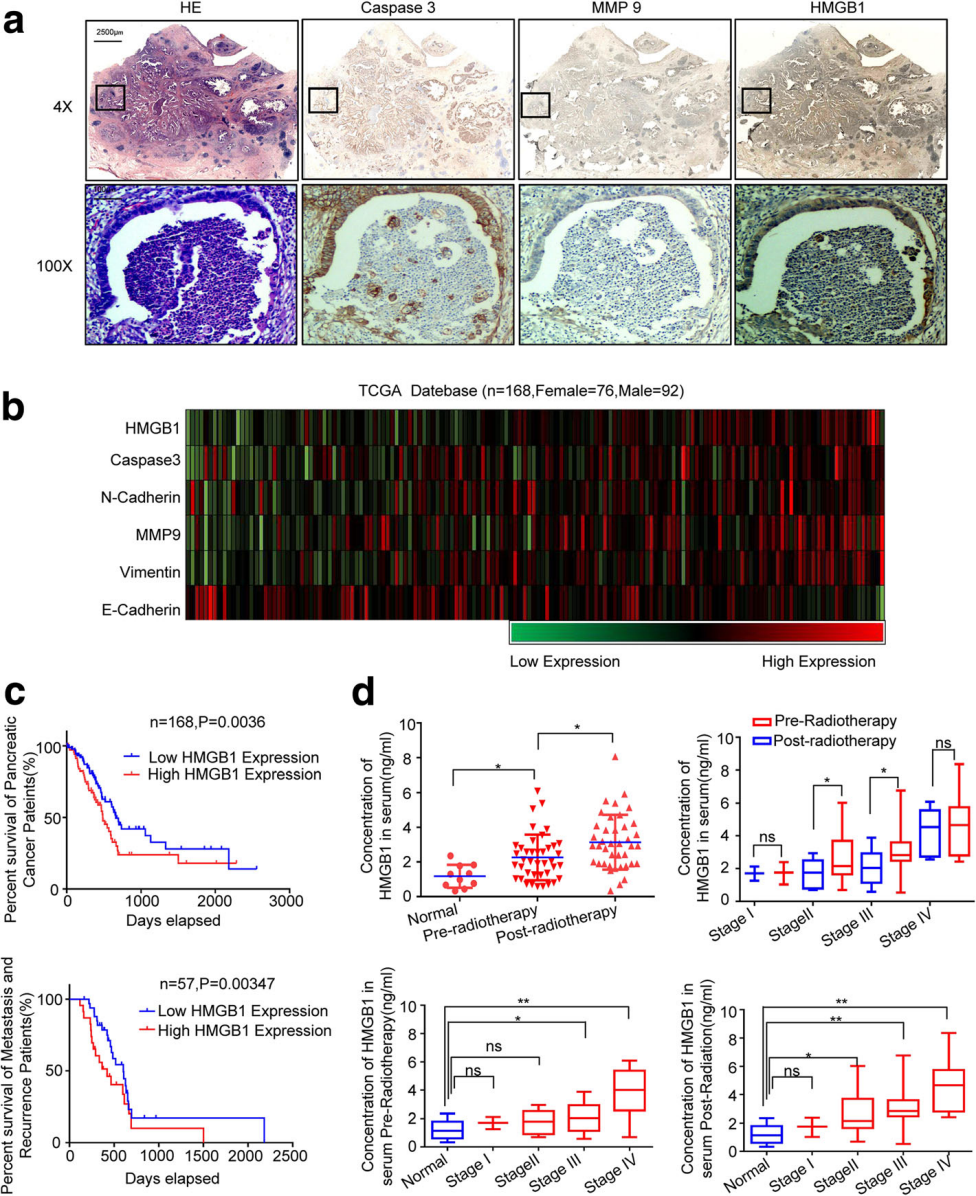
The clinical significance HMGB1 in pancreatic cancer patients. **a** IHC staining on serial sections of pancreatic carcinoma patient gross specimen showed an overlap expression of Caspase-3, MMP9, and HMGB1 in the necrosis area. The necrosis area was detected by HE staining. Caspase-3, MMP9, and HMGB1 location and expression level were accessed by IHC. Magnification: × 4 and × 10. **b** Analysis of the TCGA database indicates that the expression level of HMGB1, Caspase-3, and EMT-related proteins were highly consistent. The results are presented by heat map: *n* = 168. **c** Analysis of the TCGA database indicates that HMGB1 expression is correlated with patient survival: *n* = 84 for HMGB1-low group; and *n* =84 for HMGB1-high group. **d** The HMGB1 level in serum was higher following radiotherapy and highly related with TNM staging. *p < 0.05, **p < 0.01, ***p < 0.001

To connect our discovery to clinical application, using the pancreatic carcinoma patient tissue specimen, we performed immunehistochemical staining on serial sections to access the location and expression level of Caspase-3, a dying cancer-cell marker, MMP-9, a tumor-metastasis marker, and HMGB1. Interestingly, we noticed an overlapping expression of these markers in the hypercellular zones surrounding the necrotic foci. Caspase-3 positive staining was mainly localized in the cytoplasm (with some at the membrane), HMGB1 was mainly localized in the cytoplasm (with some at the nucleus), while MMP-9 was localized in the cytoplasm (Fig. 6a). Due to the limitations of the tissue samples, we evaluated the correlation expression of HMGB1-, Caspase-3-, and EMT-related markers (E-cadherin, N-cadherin, and vimentin) based on the information in TCGA database (https://xenabrowser.net/datapages/?cohort=TCGA%20Pancreatic%20Cancer%20(PAAD)). The heatmap revealed a linear relationship between the expression of caspase-3- and EMT-related markers with HMGB1 using TCGA protein-array data (Fig. 6b). Kaplan–Meier analysis of the TCGA data, with patients divided into high- or low-HMGB1 groups, showed a trend of lower survival, metastasis-free survival, and high local recurrence associated with high HMGB1 expression (Fig. 6c). Meanwhile, data from our hospital showed that high HMGB1 levels positively correlated with high TNM staging, distant metastasis, and radiotherapy resistance (Table 1). The serum results showed that the HMGB1 level was higher in pancreatic carcinoma following radiotherapy. Also, a high HMGB1 level was significantly associated with poor prognosis in advanced-pancreatic-cancer patients both at baseline and following radiotherapy (Fig. 6d).

## Discussion

**Fig. 7:**
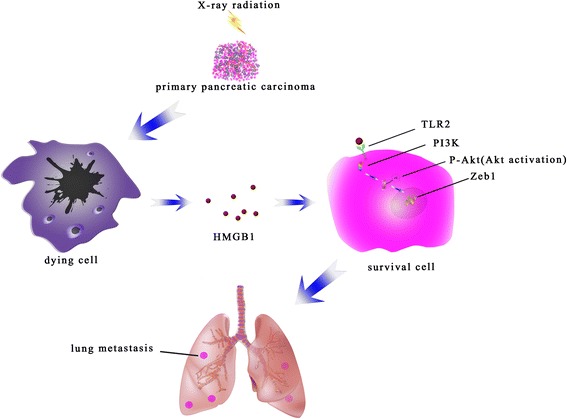
A schematic of radiotherapy induced cell death activates paracrine HMGB1-TLR2 signaling and accelerates pancreatic carcinoma metastasis. Radiotherapy induced the cancer cells’ death. HMGB1 is released by dying cells and binds specific receptor TLR2. HMGB1/TLR2 stimulates phosphorylation of PI3K/Akt and EMT in a paracrine manner, which promotes the metastasis of cancer cells to the lung

In summary, in this study we found that irradiation-induced cell death contributes to cell migration in vitro and accelerates metastasis of pancreatic carcinoma in vivo. Furthermore, dying-cell-derived HMGB1 activates the TLR2/PI3K/Akt pathways and induces the EMT program, which are important signaling events underlying radiotherapy-driven acceleration of tumor metastasis (Fig. 7).

The purpose of chemo- or radiotherapy is to induce tumor cells’ death. However, there is increasing evidence showing that irradiation can also cause various changes in the tumor microenvironment and promote tumor relapse. Dying cells can accelerate repopulation of the resident cancer cells and establish a pro-angiogenic microenvironment for tumor development [26, 27]. Our previous research has also confirmed that, if irradiation induced, the dying cells can cause pro-proliferation of the resident cancer cells in pancreatic carcinoma. Repopulation and establishment the pro-angiogenic microenvironment are critical events for the tumor metastasis. Through the in vitro and in vivo study, we first confirmed the role of dying cells in promoting the pancreatic cancer cells’ invasion and migration, which is the initial step for tumor metastasis. In the in vivo tumor model, the number of metastasis lung foci and metastasis lymph nodes detected by CT was much lower than the gross specimen. This is because CT has a low tissue resolution.

Dying cells that released chemokines and cytokines, especially the damage-associated molecular pattern (DAMP) molecules, may function in a paracrine or autocrine manner to affect each step in the metastatic cascade [28]. As a common DAMP, the HMGB1-expression level is elevated in certain cancer types. It was reported that HMGB1 released from necrotic cancer cells could enhance regrowth and metastasis of remnant colon cancer cells [29].Our results demonstrated that the level of HMGB1 was significantly elevated in both the cancer-cell cultured medium in vitro and the mice model following radiotherapy. From the clinic’s patient data, we further confirmed that HMGB1 is associated with pancreatic-carcinoma TNM staging and metastasis. These results suggested that dying-cell-derived HMGB1 plays an important role in promoting pancreatic progression. Depending on the cellular localizations, the effect of HMGB1 on cancer progression is complicated and paradoxical [14]. Kang et al. reported that intracellular HMGB1, as a novel tumor suppressor of pancreatic carcinoma, remarkably suppressed K-Ras-driven pancreatic tumorigenesis [30]. Zhang et al. found that secreted HMGB1 showed no association with TNM staging and metastasis in colorectal cancer [31]. Chen et al. demonstrated that the HMGB1–RAGE axis contributes to the migration and invasion of hepatocellular carcinoma cell lines [32]. In this study, the number of metastasis lymph nodes and lung foci was more in the N-S-treated group than the other groups. This can be explained by the N-S group including more types of chemokines and cytokines that can induce the cancer cells’ metastasis. Moreover, HMGB1-induced pancreatic cancer cells’ invasion and metastasis was mediated by the receptor for TLR2, rather than TLR4 or RAGE. This contradicts previous research that has found that RAGE was strongly expressed in human pancreatic carcinoma cells and mediated the high metastatic ability of cancer cells [33]. It has been reported that TLR2 is present in over 70% of pancreatic tumors and in cell lines from metastases, but not in normal pancreas tissue [34]. Under stress or tissue damage (the results of radiotherapy), TLR2 is the main sensor for endogenous molecules released into the extracellular compartment. These results imply a potential in developing high-affinity, tumor-targeted therapies using TLR2 following radiotherapy.

The enhanced migration and invasion ability of epithelial tumors often acquired the EMT phenotype, which is characterized by the loss of epithelial differentiation and the acquisition of a mesenchymal phenotype such as N-cadherin or vimentin. Irradiation can induce EMT to enhance the invasion and motility in breast, lung, and glioma cancer cells [9]. Many soluble factors have been reported as attending this process, such as TGF-β [35]. Our results first demonstrated that dying-cell-derived HMGB1 can induce the pancreatic cancer cells’ EMT program and further promote metastasis. EMT programs are regulated by a network of signaling pathways, including WNT, Hedgehog, ERK, and PI3K/Akt. In the present study, HMGB1/TLR2, dependent on the PI3K/Akt signaling pathways, induces pancreatic cancer cells’ EMT and metastasis. The PI3K/Akt signaling pathway is activated in pancreatic carcinoma and plays a crucial role in cell proliferation, migration, invasion, and tumor angiogenesis. The activated signaling pathway regulated the EMT-related transcription factors (Snail, Slug, Zeb1, and Twist) and induced the EMT program. Snail has always been shown to play a crucial role in irradiation-induced EMT, migration, and invasion [36]. Our results demonstrate that EMT is regulated by transcription factors Zeb1 and Slug, rather than Snail, in the presence of HMGB1. This paradoxical conclusion can be explained by the effect of EMT-related transcription factors on triggering the EMT program depending on the multiple context-dependent signals and the tumor microenvironment. Our results confirm those of previous research that high levels of Zeb1 and phosphorylated Akt(S473) in nasopharyngeal carcinoma patients are correlated with recurrence and distance metastasis in subsequent radiotherapy [37]. EMT has been shown to play an important role in the acquisition the stemness of cancer cells. Our results also confirm that rhHMGB1 could elevate the expression of CD133, which is the marker of pancreatic-cancer stem cells. The exact mechanism needs to be investigated in our future research.

## Conclusion

In summary, our results suggest that combining radiotherapy and an HMGB1 inhibitor, or targeting TLR2, may be considered a new therapeutic strategy to avoid pancreatic-carcinoma relapse and distant metastasis.

## Additional files

Additional file 1: **Figure S1.** Irradiation-induced fibroblast cell death promotes cancer-cell metastasis in vitro. (PDF 131 kb)

Additional file 2: **Figure S2.** Dying-cell-derived HMGB1 regulates tumor cell proliferation in vitro. (PDF 280 kb)

## Abbreviations

CD133: Cluster of differentiation 133
CT: Computed tomography
DAMP: Damage-associated molecular pattern.
DMEM: Dulbecco’s modified Eagle’s medium
EGF: Epidermal growth factor
EMT: Epithelial-mesenchymal transition
FBS: Fetal bovine serum
HMGB1: High-mobility group box 1
IHC: Immunohistochemical
PC: Pancreatic carcinoma
RAGE: Receptor for advanced glycation end products
TGF-β: Transforming growth factor-β
TLR: Toll-like receptors
