# Supplementary material for: Radiotherapy-induced cell death activates paracrine HMGB1-TLR2 signaling and accelerates pancreatic carcinoma metastasis
Source: J Exp Clin Cancer Res. 2018 Apr 3;37:77. doi: 10.1186/s13046-018-0726-2 (PMC5883315; doi:10.1186/s13046-018-0726-2)
Supplement: Supplementary file 1 — Figure S1. Irradiation-induced fibroblast cell death promotes cancer-cell metastasis in vitro. (PDF 131 kb) [file 13046_2018_726_MOESM1_ESM.pdf]

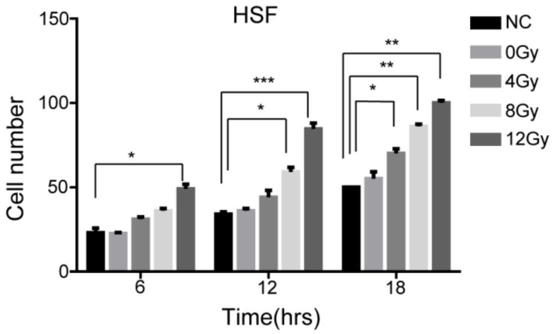

Fig. S1 Irradiation-induced fibroblast cell death promotes cancer cell metastasis in vitro. Varied-dose X-ray (0, 4, 8, and 12Gy) treated fibroblast cells seeded in the lower chamber and Panc-1 and SW1990 cells in the upper chamber co-cultured in the transwell system for the indicated time (6, 12, and 18h). Imagings were taken by electron microscope. Magnification: ×20. Experiments were repeated three times and the data were expressed as mean±SEM. * p<0.05, ** p<0.01, *** p<0.001.
