# Supplementary material for: Radiotherapy-induced cell death activates paracrine HMGB1-TLR2 signaling and accelerates pancreatic carcinoma metastasis
Source: J Exp Clin Cancer Res. 2018 Apr 3;37:77. doi: 10.1186/s13046-018-0726-2 (PMC5883315; doi:10.1186/s13046-018-0726-2)
Supplement: Supplementary file 2 — Figure S2. Dying-cell-derived HMGB1 regulates tumor cell proliferation in vitro. (PDF 280 kb) [file 13046_2018_726_MOESM2_ESM.pdf]

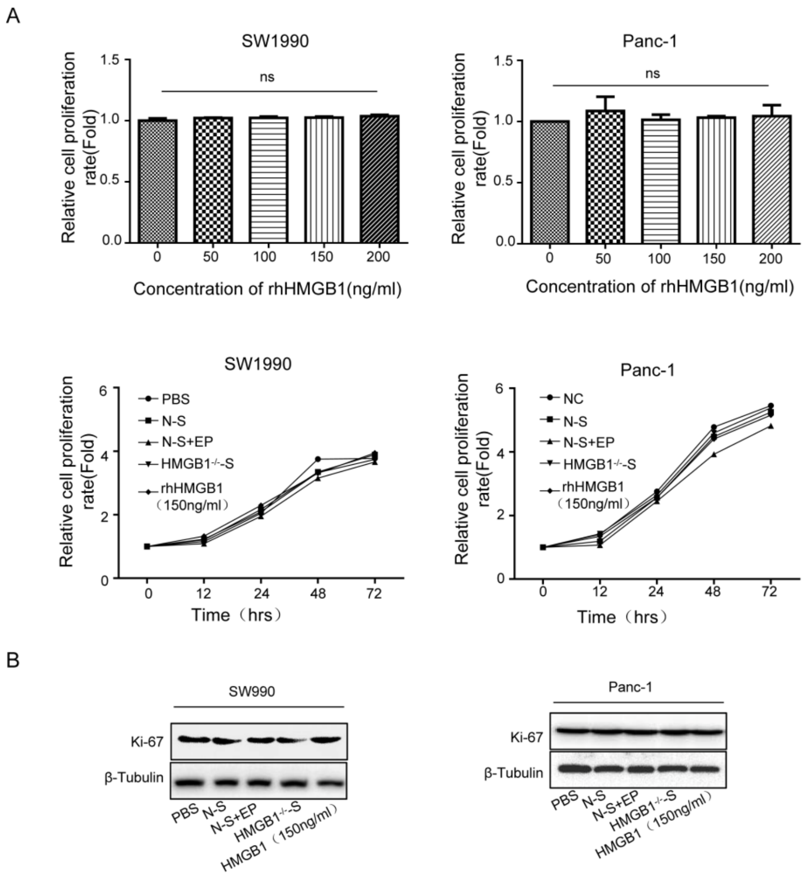

Figure S2. Dying cell-derived HMGB1 regulates tumor cell proliferation in vitro. (A) CCK8 analyzing the proliferation ability of Panc-1 and SW1990 cells following treated with N-S(supernatants from the parental cancer cells following 12Gy radiation), HMGB1$^{-/-}$-S(supernatants from the HMGBl knockdown cancer cells following 12Gy radiation), N-S+EP (HMGB1 inhibitor) and different concentrations of rhHMGB1 (50, 100, 150, 200ng/mL) for the indicated time(0, 12, 24, 48, and 72h). HMGB1 had no effect on the proliferation of cancer cells until 24h. (B) Western blot analyzing the expression of Ki67 in Panc-1 and SW1990 cells treated with PBS, N-S, HMGB1$^{-/-}$-S, N-S+EP and rhHMGB1 (150ng/mL) for 12h. β-Tubulin was a loading control. Experiments were repeated three times and the data were expressed as mean ± SEM. **P*<0.05, ***P*<0.01, ****P*<0.001.
